# Sympathetic Modulation of Neuroinflammation by Reserpine Improves Neurogenesis in ICR Mice with Traumatic Brain Injury

**DOI:** 10.3390/biomedicines14102330

**Published:** 2026-10-13

**Authors:** Natalia Ermakova, Edgar Pan, Victoria Skurikhina, Valentina Pan, Irina Zharkikh, Mariia Zhukova, Alexander Dygai, Evgenii Skurikhin

**Affiliations:** 1 Institute of General Pathology and Pathophysiology, 125315 Moscow, Russia; nejela@mail.ru (N.E.); vskurikhina@mail.ru (V.S.); valentinaglazacheva@gmail.com (V.P.); terekhina@rambler.ru (I.Z.); mashazyk@gmail.com (M.Z.); pgamrik@yandex.ru (A.D.); eskurihin@inbox.ru (E.S.); 2 Goldberg Research Institute of Pharmacology and Regenerative Medicine, 634028 Tomsk, Russia

**Keywords:** traumatic brain injury, neurogenesis, neuroinflammation, reserpine, neuronal cells, neural stem cells, regeneration

## Abstract

**Background**: Reserpine’s effect on sympathetic regulation of inflammatory cell mobilization and migration reduces neuroinflammation after traumatic brain injury (TBI). We hypothesize that an important consequence of reserpine effect may be the acceleration of brain regenerative processes. **Methods**: Neuroinflammation and the response of neuronal cells and neural stem cells in the cerebral cortex (CC) and subventricular zone (SVZ) of ICR mice after TBI and reserpine treatment were assessed using histological and immunohistochemical methods. **Results**: TBI was accompanied by neuroinflammation, cellular and tissue degeneration, neuronal cell damage, and impaired proliferation of neural stem cells in CC and SVZ. Reserpine administration inhibited neuroinflammation and reduced the severity of degenerative processes. Furthermore, an increase in the number of neural stem cells and neuronal cells was observed in the CC and SVZ. **Conclusions**: Reserpine is a promising compound for developing a new approach to TBI therapy aimed at blocking the sympathetic pathway of neuroinflammation and degeneration. This results in accelerated neuronal cell regeneration.

## 1. Introduction

Traumatic brain injury (TBI) remains a leading cause of death and long-term disability worldwide [1]. Blood–brain barrier disruption, release of damage-associated molecular patterns, and activation of microglia and astrocytes after brain injury contribute to the early neuroinflammatory and systemic inflammatory response [2,3,4]. TBI guidelines focus on controlling ischemia that develops from primary injuries and monitoring cerebral perfusion pressure [3,5]. Control of ischemia is primarily necessary to preserve and functionally restore damaged brain cells and reduce the severity of secondary damage.

Phase 3 clinical trials have evaluated the efficacy of several agents, including the glutamate receptor antagonist dexanabinol, the calcium channel blocker nimodipine, and recombinant erythropoietin. However, these approaches have shown limited efficacy in TBI [6,7,8,9].

In addition to preserving neurons damaged by injury, modern research is aimed at finding approaches to restoring lost neurons using cell or cell product therapy. Preclinical studies have revealed neuroprotective effects of human Schwann cell-derived exosomes in rats with severe penetrating brain injury [10]. Phase II clinical trials demonstrated improvement in motor and cognitive functions after autologous bone marrow mononuclear cell administration [11]. A prospective observational study to evaluate the efficacy and safety of MUSE stem cell therapy in patients with TBI is planned to begin in 2028 (NCT07326059).

Changes in systemic and local immunity after TBI are often accompanied by sympathetic hyperactivation. Catecholamine exposure contributes to metabolic stress, hemodynamic instability, and immune dysregulation [2,12,13]. For example, in a rat model of acute TBI, reserpine administration reduced the bone marrow hematopoiesis, the mobilization of inflammatory cells from the bone marrow and their homing to the brain of TBI mice. Sympathetic nervous system activation was accompanied by elevated circulating norepinephrine levels, increased PD-1 expression on CD4^+^ and CD8^+^ T-cells, and impaired IFN-γ and TNF-α production by T-cells [14]. Propranolol restored production of IFN-γ and TNF-α by T-cells in vivo. Thus, by attenuating excessive adrenergic signaling, beta-blockers may preserve cellular immunity [13]. However, these drugs have less effect on catecholamine levels, which maintains the risk of damage to brain structures.

In searching for methods to preserve and restore neuronal cells, we noted that bone marrow, as the main organ of hematopoiesis, is a source of immune cells. A previous study revealed a dependence of neuroinflammation on the activity of bone marrow hematopoiesis [15]. Reserpine is a sympatholytic agent whose main action is associated with the depletion of catecholamines. Specifically, reserpine inhibits the vesicular monoamine transporter (VMAT), blocking the reuptake of the neurotransmitter (norepinephrine) and depleting its stores in vesicles. The interaction of reserpine with VMAT initially leads to an increase in the catecholamines’ release and a subsequent decrease in their secretion [16]. Norepinephrine released under the influence of reserpine is broken down in the cytosol by monoamine oxidase [17]. This ultimately leads to a decrease in the release of catecholamines into the synaptic cleft and reduces adrenergic stimulation. The mechanism of action of reserpine is shown in Figure A1. Reserpine administration reduced bone marrow hematopoiesis and the mobilization of inflammatory cells from the bone marrow and their homing to the brain of TBI mice.

Interest in reserpine is driven by another phenomenon, perhaps more important than its effects on hematopoiesis and cell redistribution. In models of diabetes, pulmonary fibrosis, and liver cirrhosis, reserpine demonstrated regenerative effects in addition to its anti-inflammatory action [18,19,20]. In light of the above, it was suggested that reserpine administration can have a positive effect on neuronal cells in TBI.

This study aimed to evaluate the effects of reserpine on neuronal cells and neuronal progenitors of the cerebral cortex (CC) and subventricular zone (SVZ) in male ICR mice with TBI.

## 2. Materials and Methods

### 2.1. Animals

50 male ICR mice aged 12–14 weeks, obtained from the Department of Experimental Animal Clinic, Institute of General Pathology and Pathophysiology (Moscow, Russia), were used for the experiment. All animal use and studies were maintained in accordance with the European Convention for the Protection of Vertebrates and the Principles of Good Laboratory Practice (OECD, ENV/MC/CUEM (98)17, 1997), and were approved by the Ethics Committee of the Institute of General Pathology and Pathophysiology (Protocol No. 2 of 12 March 2024). The mice were housed five to six mice per cage with a 12 h light/dark cycle. Throughout the experimental period, the mice had free access to drinking water and standard rodent chow. The animals were sacrificed by CO_2_ overdose.

### 2.2. Experimental Groups and Study Design

The animals were divided into three groups. Ten mice were served as intact control. Forty animals underwent TBI and were assigned either to an untreated TBI group (*n* = 20) or to a TBI group treated with reserpine (*n* = 20). Brain tissue samples were obtained on days 7 and 21 post-TBI. Tissue damage, degenerative changes, and the number of cells expressing Ki-67, DCX, and Nestin were examined in the CC and SVZ of the right cerebral hemisphere.

### 2.3. Model of Traumatic Brain Injury

Experimental TBI was induced using a focal impact from a free-falling weight on the skull bones under inhalation anesthesia (isoflurane, Sigma-Aldrich, Saint Louis, MO, USA) [16]. Postmortem examination confirmed injury at the target location. Animals were excluded from the experiment in cases of skull fracture, impactor ricochet, or when the impact site was displaced more than 2 mm from the bregma. The day of TBI induction was designated as day 0.

### 2.4. Reserpine

Reserpine (Sigma-Aldrich, Saint Louis, MO, USA) is an indole alkaloid derived from *Rauvolfia serpentina*. Its sympatholytic effect is associated with depletion of catecholamines from presynaptic nerve terminals due to their release into the cytosol with subsequent degradation by monoamine oxidase.

### 2.5. Administration of Reserpine

Reserpine was dissolved in physiological saline and administered intraperitoneally in a volume of 0.1 mL per mice on post-injury days 3–7. The initial dose was 1.0 mg/kg, whereas the subsequent injections were 0.1 mg/kg. TBI and intact mice were administered an equivalent volume of physiological saline. The doses and regimens for administering reserpine were selected based on our previous research [15].

### 2.6. Morphometric Examination

#### 2.6.1. Preparation of Histological Samples

Histological and Immunohistochemical (IHC) analysis of the CC and SVZ in the right cerebral hemisphere was conducted in the damaged area (from +0.50 to −0.10 mm relative to the bregma). For this purpose, the brains were extracted and placed in 10% formaldehyde solution, followed by standard cryo-preparation using sucrose solutions of increasing concentration in formaldehyde-based buffer. The tissue was frozen in liquid nitrogen vapor and stored at −80 °C. Cryosections with a thickness of 10 microns were prepared using a cryostat (MCM-3500, MT Point, Saint-Petersburg, Russia).

#### 2.6.2. Histological Examination

Histological sections were stained with hematoxylin and eosin using standard protocols as previously shown. Cryosections were washed in distilled water and stained sequentially with Carrazzi’s hematoxylin, with differentiation in Scott’s Tap Water Substitute and an aqueous eosin solution. After staining, the sections were washed and dehydrated in isopropanol and mounted in mounting medium [15,21,22].

Tissue and cellular edema, vascular structural disorders, inflammatory infiltration, the presence of hemorrhages, the fullness of blood vessels, the presence of neurons with hypochromic and hyperchromic nuclei, pyknotic nuclei, vacuolated cells, and dividing neuroglial cells were evaluated in the damaged area. Representative images of the above changes are shown in Figure A2. For each animal, 5 sections with 5 fields in each were analyzed. A semi-quantitative assessment of tissue changes was made according to the method of Arslan E. et al. (2026) with our modification [15,21,23].

In particular, each parameter was assessed on a 6-point scale:

- 0 points: Absent (normal);
- 1 point: Minimal involvement, characterized by single or weakly expressed focal changes;
- 2 points: Mild involvement, characterized by a few focal changes with low frequency;
- 3 points: Moderate involvement, characterized by multiple and clearly expressed changes;
- 4 points: Marked involvement, characterized by widespread and/or intensely expressed changes;
- 5 points: Severe involvement, characterized by extensive, sharply expressed changes.

#### 2.6.3. Immunohistochemical Examination

Specific cellular markers were identified using rabbit polyclonal antibodies against doublecortin (GB114319, Rabbit Anti-Doublecortin; Servicebio Technology, Wuhan, China), mouse monoclonal antibodies against Ki-67 (GB121141, Mouse Anti-Ki-67; Servicebio Technology, Wuhan, China), and mouse monoclonal antibodies against Nestin (GB12160, Mouse Anti-Nestin; Servicebio Technology, Wuhan, China).

Micrographs of brain samples stained with IHC were obtained using an inverted fluorescence microscope Nexcope NIB900 (Ningbo Yongxin Optics Co., Ltd., Ningbo, China) equipped with a Dhyana 400D camera (Tucsen Photonics Co., Ltd., Fuzhou, China). Image analysis was performed using ImageJ software (version 2.16.0/1.54p; NIH). For each staining, microphotographs of 5 brain sections were taken. Subsequently, in 3 fields of view (200 × 200 μm area each), we quantified the total number of nuclei and the number of cells expressing doublecortin (DCX), the marker of neuronal progenitor cells, Ki-67, a proliferative activity marker, and Nestin, a neuroepithelial progenitor cell marker. In addition, the content of cells co-expressing DCX/Ki-67 and DCX/Nestin was assessed.

### 2.7. Statistical Analysis

Statistical analysis was performed using SPSS (version 15.0, SPSS Inc., Chicago, IL, USA). Data were analyzed and presented as M ± m. The normality of data distribution was assessed using the Shapiro–Wilk test. The significance was evaluated by the nonparametric Kraskel-Wallis H test, PostHoc analysis was performed using the nonparametric Mann–Whitney U test. A *p*-value of less than 0.05 (by two-tailed testing) was considered an indicator of statistical significance. For multiple comparisons, the Bonferroni correction was applied to adjust the *p*-value (*p* < 0.01).

## 3. Results

### 3.1. Morphology of the Mouse Brain After TBI

#### 3.1.1. Histological Examination

The morphological disturbance in the brain found in this study was largely consistent with what we observed previously [15]. Analysis of mouse brain sections stained with hematoxylin and eosin revealed inflammatory infiltration, vascular congestion, and hemorrhages against a background of compromised vascular wall integrity in CC and SVZ of the right hemisphere at the injury sites (days 7 and 21). In the damaged areas, edema, congestion, and degenerative changes were observed, and a glial scar was formed (Table 1). The representative micrographs of the brain of male ICR mice are shown in Figure A3.

#### 3.1.2. Immunohistochemical Examination

Cerebral cortex. The reduction in the number of DAPI-stained cells was observed in the CC of TBI mice on day 7 post-injury. In addition, the number of cells expressing DCX, as well as cells co-expressing Nestin and DCX, decreased in the same group on days 7 and 21 post-TBI, compared to intact animals (Figure 1, Table 2).

Subventricular zone. Increased expression of Nestin and DCX was detected in the SVZ of TBI mice compared to intact animals at day 7 post-TBI. At day 21, DCX and Nestin expression was also higher in mice of the same group compared to intact mice (Figure 2, Table 3).

### 3.2. Reserpine Caused Changes in Brain Morphology in TBI

#### 3.2.1. Histological Examination

Analysis of hematoxylin and eosin-stained brain sections showed that reserpine treatment reduced inflammatory infiltration in the MC and SVZ of treated TBI mice compared with untreated mice (Table 1, Figure A3). In addition, a decrease in the severity of degenerative changes and a reduction in glial scar formation were observed.

#### 3.2.2. Immunohistochemical Examination

Cerebral cortex. Reserpine had no significant effect on the number of Nestin^+^ or DCX^+^ cells in the CC of reserpine-treated TBI mice relative to untreated TBI mice throughout the observation period (Figure 1, Table 2). However, the number of DAPI-stained cells increased on day 7. The number of Ki-67^+^ cells in the CC of treated mice decreased, while the number of DCX^+^Ki67^+^ cells, on the contrary, increased relative to untreated animals on day 21 (Figure 1, Table 2).

Subventricular zone. The SVZ of reserpine-treated TBI mice had significantly more DAPI-stained cells at day 7. At the same time, the number of DCX^+^, Ki-67^+^, and DCX^+^Ki-67^+^ cells decreased (Figure 2, Table 3). The number of DAPI-stained cells in the SVZ of reserpine-treated animals increased on day 21, as well as DCX^+^Ki-67^+^ cells. The number of DCX^+^, Nestin^+^, and Nestin^+^DCX^+^ cells was reduced significantly.

## 4. Discussion

Surgical treatment and conservative therapy of TBI are aimed at maintaining vital functions and preventing secondary brain damage. The issue of correcting neuroinflammation, which greatly increases the risk of developing Parkinson’s disease and Alzheimer’s disease, remains unresolved. Current approaches to treating TBI have limited effectiveness against neuroinflammation.

Experimental approaches aimed at combating neuroinflammation, cerebral edema, or providing neuroprotection include TNF-alpha blockade [24], neurokinin-1 receptor antagonism [25], and modulation of immunometabolism [26]. Modulation of regulatory T-cell function is an innovative preclinical strategy [27]. In a mouse model of contusional TBI, intranasal anti-CD3 therapy increased the number of IL-10-producing regulatory T-cells, reduced pathological microglial inflammation, normalized microglial phagocytic function, and improved neurological condition [28]. This approach is particularly relevant for combating chronic neuroinflammation in TBI.

Cell therapy is a promising approach to the TBI treatment. Several clinical studies assessed the efficacy and safety of the infusion of autologous bone marrow mononuclear cells and mesenchymal stem cells in patients with TBI (NCT01851083, NCT01575470, NCT02525432, NCT02028104, NCT00254722, NCT06146062) [10,29,30]. TBI is often accompanied by sympathetic hyperactivation. Excessive exposure to catecholamines contributes to metabolic stress, hemodynamic instability, and immune dysregulation [2,13,14]. In this regard, it is necessary to search for strategies aimed at combating post-traumatic neuroinflammation based on the correction of sympathetic regulation. Prospects of beta-blocker administration to correct sympathetic hyperactivation are being studied (NCT06569212) [31]. In adults with acute TBI, beta-blocker administration improved neurological outcome and was associated with lower mortality rates and shorter duration of mechanical ventilation [32]. Propranolol prevented impairment of IFN-γ and TNF-α production by T-cells associated with increased circulating norepinephrine levels in a rat model of acute TBI [14].

In a previous study, we proposed an experimental approach to combat neuroinflammation in TBI-injured mice based on reserpine administration [15]. As is known, reserpine effects are based on the accelerated release of catecholamines from presynaptic nerve endings and their subsequent inactivation by monoamine oxidase. This is how reserpine’s action differs from that of adrenergic blockers, whose effects are mediated by receptor mechanisms. Consequently, adrenoblocker administration do not exclude the involvement of a significant amount of stored catecholamines in the mechanism of tissue damage. According to our data, reserpine reduced bone marrow hematopoiesis activity, disrupted the mobilization of inflammatory cells from the bone marrow into the blood, and their migration to damaged tissue. Thus, the sympatholytic agent reduced neuroinflammation after TBI. A similar effect of reserpine has previously been observed in animal models of diabetes mellitus, pulmonary fibrosis, and liver cirrhosis [18,19,20]. It is noteworthy that, in addition to successfully combating inflammation, reserpine improved regeneration of the pancreas, lungs, and liver. The results of reserpine administration, such as accelerated regeneration in models of diabetes mellitus, pulmonary fibrosis, and liver cirrhosis, seemed important to us for the development of experimental therapy for post-traumatic neuroinflammation.

To assess the regenerative potential of reserpine, the content of DAPI-stained cells, as well as the expression of DCX, Nestin, and Ki-67, characterizing neurogenesis, in the damaged area of the CC of TBI mice was initially studied. The number of nucleated cells in the damaged area of the CC of TBI mice was reduced relative to the values in intact mice on day 7 with their number restored on day 21 (Figure 1, Table 2). In the damaged CC, the expression of DCX (a marker of neurogenesis) and Nestin (a marker of neuroepithelial progenitor cell) was reduced, and the number of cells co-expressing Nestin and DCX decreased. Co-expression of Nestin and DCX is believed to be characteristic of neural stem cells, which determine the intensity of neurogenesis. In this regard, the presented data allowed us to conclude that the intensity of neurogenesis in the area of the CC injury decreases. We believe that the observed increase in DAPI-stained cells in the injury area is associated with elevated in the number of inflammatory cells, both resident and those migrating from the periphery [15]. Thus, a possible basis for the disruption of neurogenesis in the area of the CC injury is the inhibitory effect of molecular and cellular inflammatory factors on the neural stem cell population.

According to modern concepts, the SVZ of the brain is a niche for neural stem cells. The number of nucleated cells in the SVZ of TBI mice increased relative to intact mice on day 21 (Figure 2, Table 3). This is partly explained by inflammatory infiltration. In contrast to the CC, Nestin and DCX expression increased in the SVZ of TBI mice compared to intact animals (days 7 and 21). This suggests active neurogenesis in the SVZ associated with neural stem cells. Apparently, the observed enrichment of SVZ neuronal cells (DCX^+^) was the result of differentiation of neuronal stem cells (Nestin^+^) (Figure 2, Table 3). In this case, a decrease in the number of DCX^+^ cells in the CC against the background of an increase in their number in the SVZ may be a consequence of a disruption in the migration of neuronal cells to the damaged area. The results of this IHC study, combined with previous data, confirm the involvement of SVZ neural stem cells in post-traumatic neurogenesis. However, neuroinflammation hinders the realization of the regenerative potential of neural stem cells. A possible way to accelerate neurogenesis is through correcting the sympatholytic mechanism of neuroinflammation regulation.

Reserpine changed post-traumatic neurogenesis. On day 7, a decrease in the number of DCX^+^ and DCX^+^Ki-67^+^ cells was observed in the SVZ of treated TBI mice compared to untreated animals (Figure 1 and Figure 2, Table 2 and Table 3). On day 21, an increase in the number of DCX^+^Ki-67^+^ cells was observed in both the CC and the SVZ. This is probably due to a decrease in the migration of inflammatory cells into the brain [16] and the formation of conditions for the migration and proliferation of neuronal cells. A decrease in the number of Nestin^+^DCX^+^ cells in the SVZ during therapy may indicate the differentiation of neural stem cells. Another possible mechanism of reserpine may be associated with the blockade of the inhibitory effect of catecholamines on immature and maturing neuronal cells [32].

The population of neural stem cells is quite heterogeneous [33]. Other markers of neuronal stem cells are known, such as PRC2, NeuroD1, AKH, Sox family proteins, etc. [34,35]. The lack of data on their expression in TBI largely limits the understanding of the mechanisms involved in the reserpine effects. In this study, it was important to evaluate neurogenesis against the background of reduced neuroinflammation after reserpine administration. We believe this approach offers opportunities to modulate neural stem cell activity and stimulate the regeneration of damaged brain tissue.

## 5. Conclusions

In this study, we demonstrated the effect of reserpine on neurogenesis in ICR mice after experimental TBI. Experimental reserpine therapy showed a positive effect on neurogenesis in TBI. The regenerative effect of reserpine is based on improving the migration of newly formed neuronal cells, including actively dividing ones, from the SVZ to the CC. At the same time, an acceleration of neural stem cell differentiation in the SVZ cannot be excluded. Importantly, by altering the systemic balance of catecholamines, reserpine helps reduce neuroinflammation. This creates the conditions for restoring the regenerative capacity of neural stem cells. We believe that the obtained data expand our understanding of the role of sympathetic regulation in neurogenesis after TBI. This approach may form the basis for the development of new methods aimed at improving neurogeneration after brain injury. In this study, it was important to evaluate neurogenesis against the background of reserpine’s reduction in neuroinflammation. In our view, reserpine can be considered a therapeutic agent for targeting neural stem cells to stimulate brain regeneration of injured brain tissue.

However, TBI can be accompanied by the development of delirium and episodes of psychosis, which can be caused by neuroinflammation, mediator imbalance, and other factors [36,37]. While some agents (beta-blockers, antidepressants, antipsychotics, and antipsychotics) are known to influence the severity and incidence of psychological disorders in TBI, data on the effect of reserpine are limited [37,38]. Consequently, further animal studies and carefully designed clinical trials are needed to evaluate the effects of reserpine on the severity of psychological disorders after TBI.

## Figures and Tables

**Figure 1:**
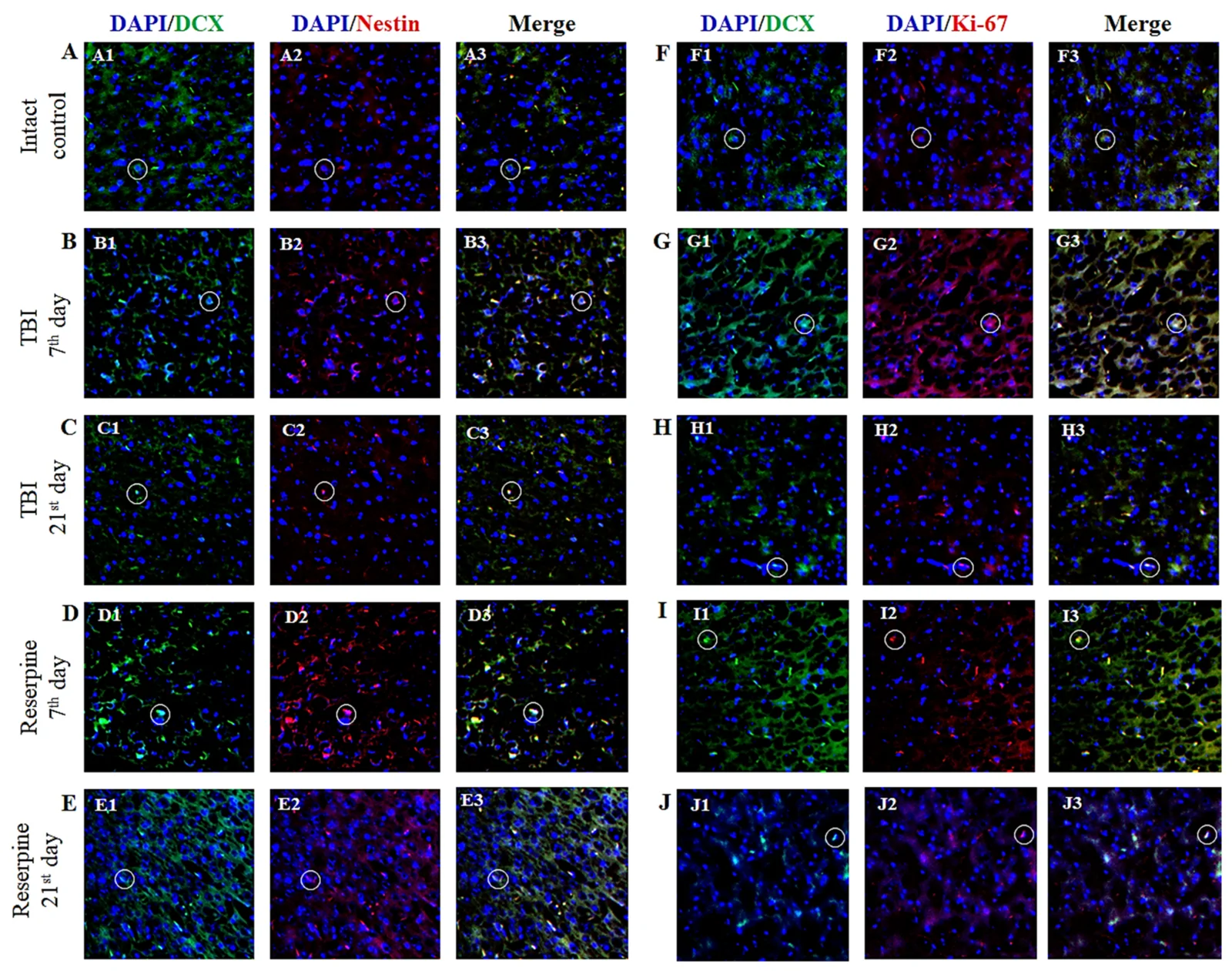
Representative micrographs of the cerebral cortex obtained from (**A**,**F**) intact mice; (**B**,**G**) TBI mice, day 7; (**C**,**H**) TBI mice, day 21; (**D**,**I**) TBI mice treated with reserpine, day 7 and (**E**,**J**) day 21. Samples were stained with antibodies specific to (**A1**,**B1**,**C1**,**D1**,**E1**,**F1**,**G1**,**H1**,**I1**,**J1**) DCX (green), (**A2**,**B2**,**C2**,**D2**,**E2**) Nestin (red), (**F2**,**G2**,**H2**,**I2**,**J2**) Ki-67 (red). DAPI (blue) was used to identify cell nuclei. (**A3**,**B3**,**C3**,**D3**,**E3**,**F3**,**G3**,**H3**,**I3**,**J3**) (Merge) Composite image using all three colors. ×100 images. The white circle represents the area of staining for the DCX, Ki-67 or Nestin markers.

**Figure 2:**
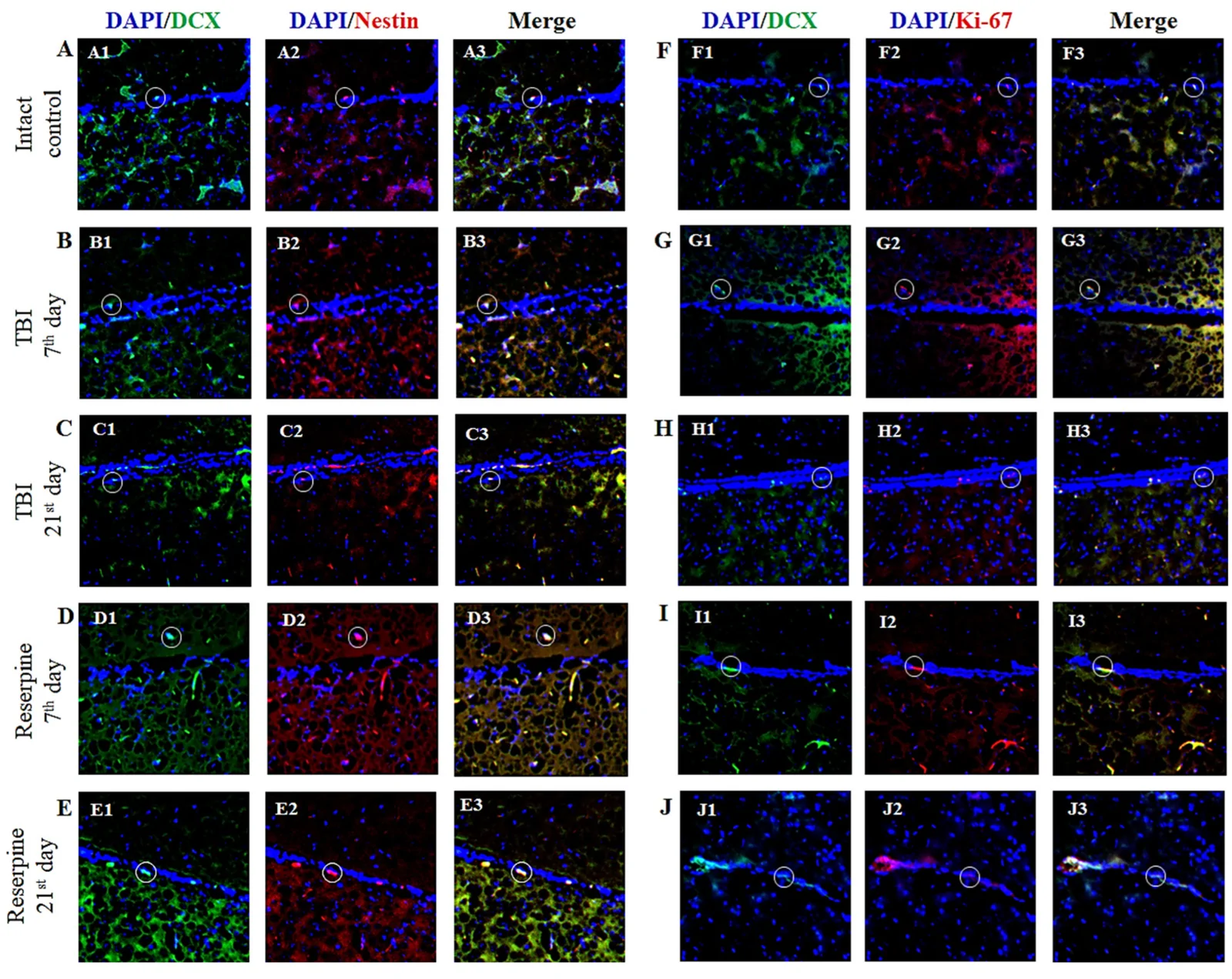
Representative micrographs of the subventricular zone obtained from (**A**,**F**) intact mice; (**B**,**G**) TBI mice, day 7; (**C**,**H**) TBI mice, day 21; (**D**,**I**) TBI mice treated with reserpine, day 7 and (**E**,**J**) day 21. Samples were stained with antibodies specific to (**A1**,**B1**,**C1**,**D1**,**E1**,**F1**,**G1**,**H1**,**I1**,**J1**) DCX (green), (**A2**,**B2**,**C2**,**D2**,**E2**) Nestin (red), (**F2**,**G2**,**H2**,**I2**,**J2**) Ki-67 (red). DAPI (blue) was used to identify cell nuclei. (**A3**,**B3**,**C3**,**D3**,**E3**,**F3**,**G3**,**H3**,**I3**,**J3**) (Merge) Composite image using all three colors. ×100 images. The white circle represents the area of staining for the DCX, Ki-67 or Nestin markers.

**Table 1:** Morphometric disturbance in the brain of male ICR mice on days 7 and 21 post-TBI and after reserpine therapy, scores (M ± m).

	Intact	TBI 7th Day	TBI 21st Day	Reserpine 7th Day	Reserpine 21st Day
Tissue edema	0.00 ± 0.00	1.18 ± 0.20 *	0.93 ± 0.16 *	1.05 ± 0.18	0.49 ± 0.12
Cellular edema	0.67 ± 0.11	2.54 ± 0.10 *	2.88 ± 0.07 *	3.08 ± 0.10 •	1.98 ± 0.09 •
Hyperchromic nuclei	0.73 ± 0.12	2.85 ± 0.14 *	2.50 ± 0.10 *	2.77 ± 0.14	1.64 ± 0.13 •
Pyknotic nuclei	0.94 ± 0.10	2.74 ± 0.15 *	2.43 ± 0.08 *	2.92 ± 0.12	1.67 ± 0.13 •
Vacuolated cells	1.61 ± 0.14	4.56 ± 0.13 *	3.93 ± 0.06 *	4.79 ± 0.07	3.18 ± 0.10 •
Astrogliosis	0.42 ± 0.10	2.85 ± 0.12 *	2.98 ± 0.11 *	0.90 ± 0.12 •	1.98 ± 0.11 •
Inflammatory infiltration	0.00 ± 0.00	2.00 ± 0.13 *	2.57 ± 0.08 *	1.13 ± 0.11 •	1.69 ± 0.16 •
Hemorrhages	0.06 ± 0.04	2.00 ± 0.30 *	1.36 ± 0.25 *	1.54 ± 0.23	0.73 ± 0.15
Vascular congestion	0.18 ± 0.07	2.87 ± 0.16 *	1.81 ± 0.16 *	2.15 ± 0.14 •	1.27 ± 0.16

* Significance of difference compared with intact control (*p* < 0.01 with the Bonferroni correction). • Significance of difference compared with TBI (*p* < 0.01 with the Bonferroni correction).

**Table 2:** The count of DCX^+^, Nestin^+^ and Ki-67^+^ cells in the cerebral cortex of male ICR mice at days 7 and 21 post-TBI (M ± m).

	Intact Control	TBI 7th Day	TBI 21st Day	Reserpine 7th Day	Reserpine 21st Day
Total nuclei number	184.10 ± 6.98	134.43 ± 6.37 *	205.57 ± 5.14	158.43 ± 5.75 •	208.75 ± 6.79
DCX^+^ cells	38.19 ± 2.92	22.97 ± 2.24 *	26.90 ± 3.09 *	18.63 ± 1.77	30.96 ± 2.10 •
Nestin^+^ cells	38.55 ± 2.72	30.47 ± 2.49	29.38 ± 1.58	31.27 ± 2.73	33.71 ± 1.70
Ki-67^+^ cells	29.40 ± 1.93	29.00 ± 3.47	33.11 ± 1.21	31.22 ± 2.09	19.22 ± 0.57 •
Nestin^+^DCX^+^ cells	23.76 ± 1.94	16.37 ± 1.86 *	15.57 ± 1.08 *	18.63 ± 1.53	19.04 ± 1.35
DCX^+^Ki-67^+^ cells	14.40 ± 0.91	15.00 ± 1.69	9.67 ± 0.17 *	12.89 ± 1.84	14.67 ± 0.33 •

* Significance of difference compared with intact control (*p* < 0.01 with the Bonferroni correction). • Significance of difference compared with TBI (*p* < 0.01 with the Bonferroni correction).

**Table 3:** The count of DCX^+^, Nestin^+^ and Ki-67^+^ cells in the subventricular zone of male ICR mice at days 7 and 21 post-TBI (M ± m).

	Intact Control	TBI 7th Day	TBI 21st Day	Reserpine 7th Day	Reserpine 21st Day
Total nuclei number	387.04 ± 19.96	357.47 ± 14.14	466.08 ± 27.20 *	441.47 ± 28.09 •	529.76 ± 38.01 •
DCX^+^ cells	75.28 ± 3.64	171.22 ± 12.32 *	119.95 ± 7.80 *	93.83 ± 9.70 •	80.67 ± 9.07 •
Nestin^+^ cells	60.21 ± 3.71	96.22 ± 5.69 *	72.62 ± 4.44 *	77.77 ± 6.80	43.30 ± 3.47 •
Ki-67^+^ cells	78.70 ± 9.10	56.95 ± 3.25 *	72.58 ± 3.18 *	36.00 ± 1.59 •	77.17 ± 5.51
Nestin^+^DCX^+^ cells	22.72 ± 1.80	25.60 ± 1.52	26.00 ± 2.02	22.20 ± 2.24	12.85 ± 0.90 •
DCX^+^Ki-67^+^ cells	49.18 ± 8.38	24.69 ± 1.46 *	46.53 ± 2.75	19.58 ± 1.34 •	58.94 ± 3.50 •

* Significance of difference compared with intact control (*p* < 0.01 with the Bonferroni correction). • Significance of difference compared with TBI (*p* < 0.01 with the Bonferroni correction).

**Figure A1:**
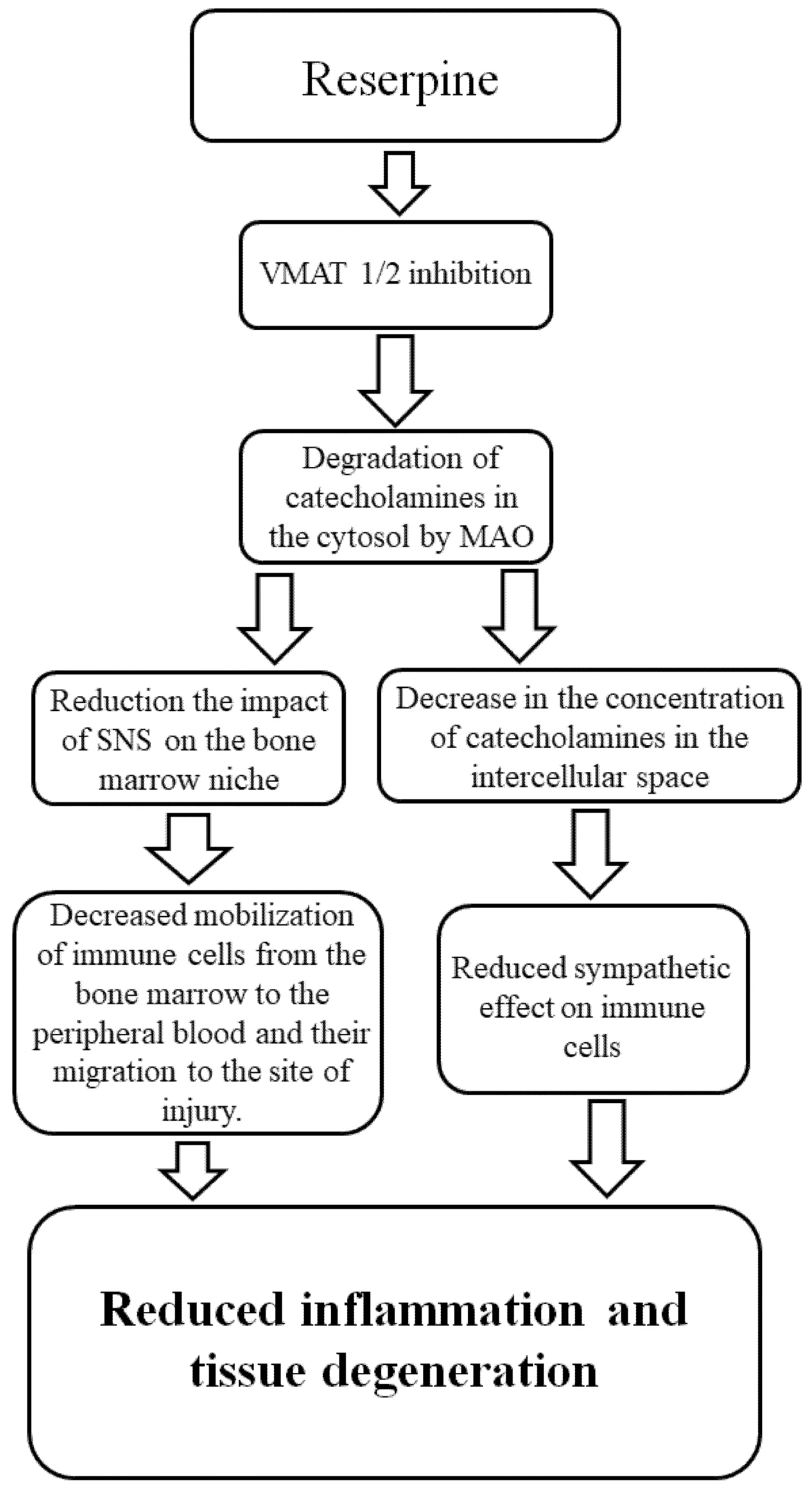
The mechanism of action of reserpine on inflammation. The mechanism is mediated through the attenuation of the sympathetic nervous system’s (SNS) influence on the bone marrow and immune cells. This occurs via the inhibition of vesicular monoamine transporters 1 and 2 (VMAT 1/2), leading to subsequent degradation of catecholamines in the cytosol by monoamine oxidase (MAO). The resultant decrease in catecholamine concentration in the intercellular space reduces the sympathetic tone on the bone marrow niche and immune cells. This ultimately diminishes the mobilization of immune cells from the bone marrow to the peripheral blood and their migration to the injury site.

**Figure A2:**
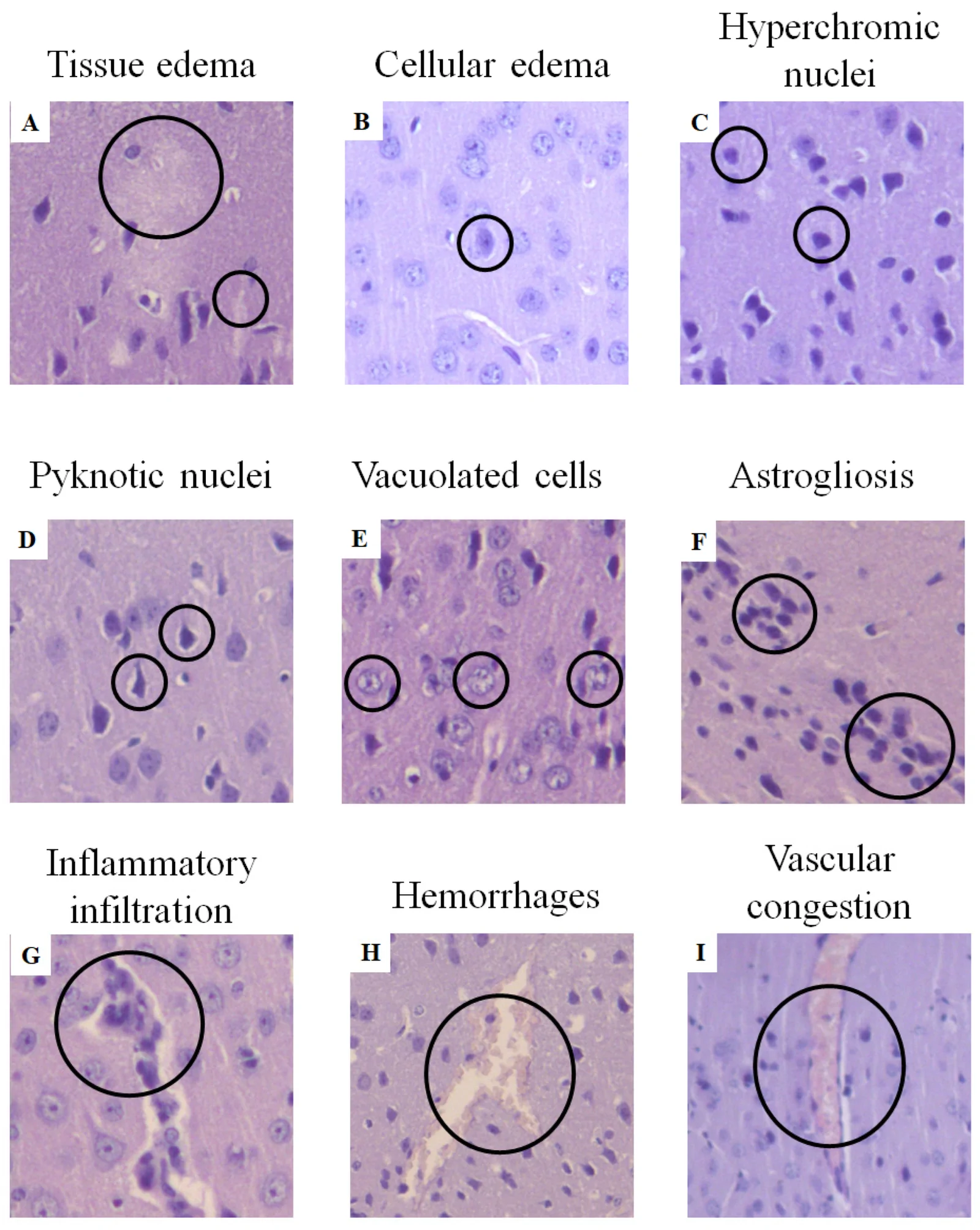
Representative micrographs of the histopathological features in the brain of male ICR mice. (**A**) Tissue edema; (**B**) cellular edema; (**C**) hyperchromic nuclei; (**D**) pyknotic nuclei; (**E**) vacuolated cells; (**F**) astrogliosis; (**G**) inflammatory infiltration; (**H**) hemorrhages; (**I**) vascular congestion. Tissue stained with hematoxylin and eosin, ×200. Note: the black circle represents the parameters that are being evaluated.

**Figure A3:**
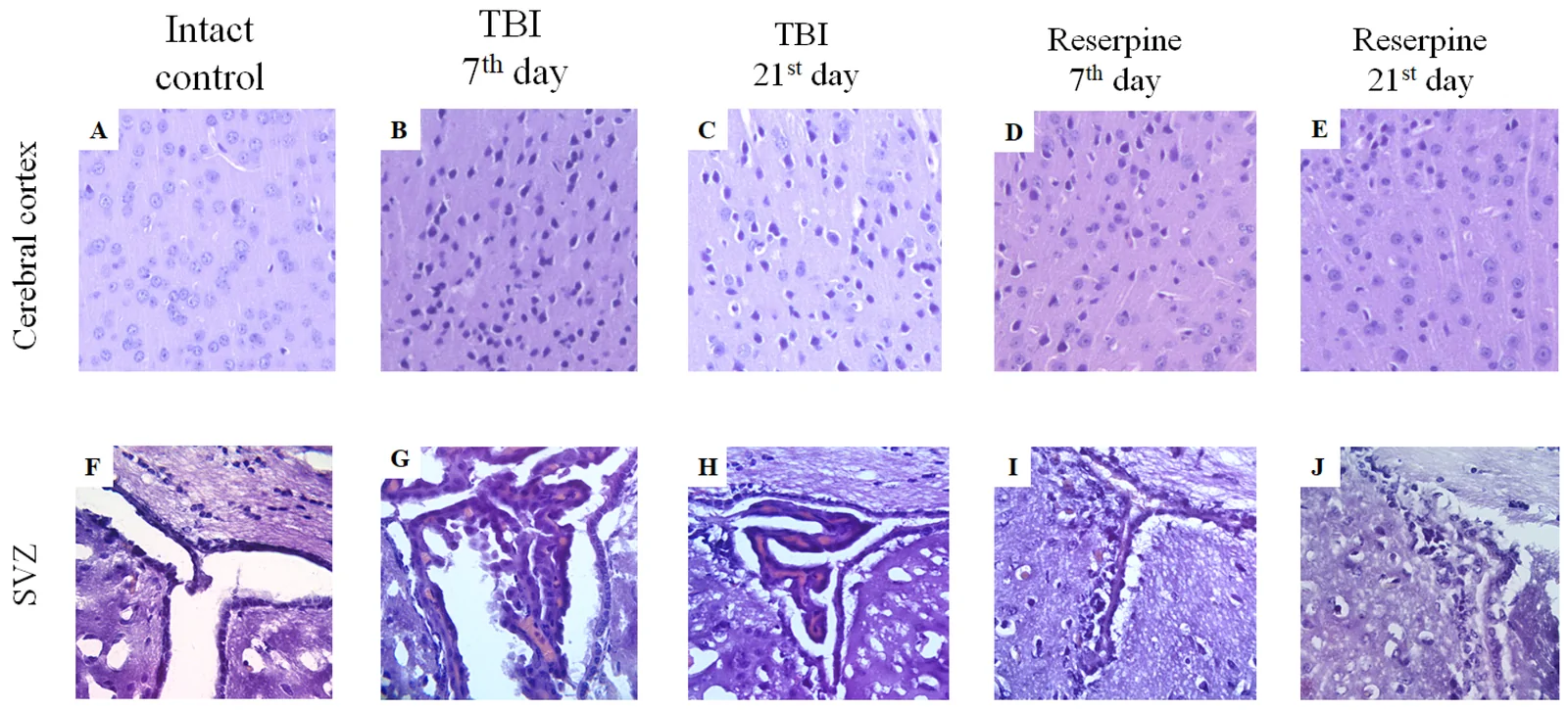
Representative micrographs of the cerebral cortex (**A**–**E**) and subventricular zone (**F**–**J**) of the brain of male ICR mice at 7 and 21 days post-TBI. (**A**,**F**) Intact control mice; (**B**,**G**) TBI mice, day 7 post-injury; (**C**,**H**) TBI mice, day 21 post-injury; (**D**,**I**) reserpine-treated TBI mice, day 7 post-injury; (**E**,**J**) reserpine-treated TBI mice, day 21 post-injury. Tissue stained with hematoxylin and eosin, ×200.

## Data Availability

The data presented in this publication can be accessed by contacting the corresponding author, subject to a reasonable request.

## Abbreviations

The following abbreviations are used in this manuscript:

CC	Cerebral Cortex
DCX	Doublecortin
SVZ	Subventricular Zone
TBI	Traumatic Brain Injury
